# Jasmonate-dependent induction of polyphenol oxidase activity in tomato foliage is important for defense against *Spodoptera exigua* but not against *Manduca sexta*

**DOI:** 10.1186/s12870-014-0257-8

**Published:** 2014-09-27

**Authors:** Marko Bosch, Sonja Berger, Andreas Schaller, Annick Stintzi

**Affiliations:** Institute of Plant Physiology and Biotechnology, University of Hohenheim (260), 70593 Stuttgart, Germany

**Keywords:** Generalist and specialist herbivores, Glucose oxidase, Insect resistance, Jasmonic acid, Oxophytodienoic acid, Plant defense, Polyphenol oxidase, Oral secretions, Terpenes

## Abstract

**Background:**

Jasmonates are involved in plant defense, participating in the timely induction of defense responses against insect herbivores from different feeding guilds and with different degrees of host specialization. It is less clear to what extent the induction of plant defense is controlled by different members of the jasmonate family and how specificity of the response is achieved. Using transgenic plants blocked in jasmonic acid (JA) biosynthesis, we previously showed that JA is required for the formation of glandular trichomes and trichome-borne metabolites as constitutive defense traits in tomato, affecting oviposition and feeding behavior of the specialist *Manduca sexta*. In contrast, JA was not required for the local induction of defense gene expression after wounding. In JA-deficient plants, the JA precursor oxophytodienoic acid (OPDA) substituted as a regulator of defense gene expression maintaining considerable resistance against *M. sexta* larvae. In this study, we investigate the contribution of JA and OPDA to defense against the generalist herbivore *Spodoptera exigua*.

**Results:**

*S. exigua* preferred JA-deficient over wild-type tomato plants as a host for both oviposition and feeding. Feeding preference for JA-deficient plants was caused by constitutively reduced levels of repellent terpenes. Growth and development of the larvae, on the other hand, were controlled by additional JA-dependent defense traits, including the JA-mediated induction of foliar polyphenol oxidase (PPO) activity. PPO induction was more pronounced after *S. exigua* herbivory as compared to mechanical wounding or *M. sexta* feeding. The difference was attributed to an elicitor exclusively present in *S. exigua* oral secretions.

**Conclusions:**

The behavior of *M. sexta* and *S. exigua* during oviposition and feeding is controlled by constitutive JA/JA-Ile-dependent defense traits involving mono- and sesquiterpenes in both species, and *cis*-3-hexenal as an additional chemical cue for *M. sexta*. The requirement of jasmonates for resistance of tomato plants against caterpillar feeding differs for the two species. While the OPDA-mediated induction of local defense is sufficient to restrict growth and development of *M. sexta* larvae in absence of JA/JA-Ile, defense against *S. exigua* relied on additional JA/JA-Ile dependent factors, including the induction of foliar polyphenol oxidase activity in response to *S. exigua* oral secretions.

## Background

Some 350 million years of common history led to the diversification and species richness of present-day flowering plants and phytophagous insects. The joint success of these two closely interacting taxonomic groups has been explained by co-evolution [1-4]. In adaptation to the selection pressure exerted by herbivores, plants evolved constitutive and inducible defense systems that appear to be tailored specifically to different aggressors [5,6]. They include direct defenses such as anti-nutritive proteins, repellant or toxic secondary metabolites, and morphological features such as thorns, prickles or trichomes [7,8]. In addition, plants produce volatile compounds and nectar rewards to attract natural enemies of their pests resulting in indirect defense [9-11].

Insect herbivores vary greatly with respect to their ability to cope with multi-faceted plant defense and this variability largely determines host range and diet breadth of the insect [12,13]. As generalists, polyphagous insects tolerate a wide array of plant defense traits and they may overcome induced defense by manipulating conserved signaling mechanisms that are commonly found in all plants. With increasing specialization, oligo- and monophagous insects appear to have lost the ability to exploit many different plant species but evolved mechanisms to cope with the particular defense traits of their host, and to even manipulate host characteristics to their own benefit [4,14]. As a corollary of the generalist-specialist paradigm, it was assumed that generalist and specialist herbivores would interact with their host plants in distinct and predictable ways. However, this assumption has recently been challenged [14]: while plants clearly show different responses to insects from different feeding guilds, the evidence linking differences in plant responses to the degree of insect specialization is less convincing [5,15-19].

The open question of whether plant responses are divided along the specialist-generalist dichotomy notwithstanding, there is no doubt that plants respond differently to different insects, implying the existence of specific stimuli and recognition systems. Some plant responses are triggered by the loss of tissue integrity as it is caused by herbivory or by mechanical wounding [8]. These responses do not rely on the presence of the herbivore but rather depend on the recognition of damaged-self mediated by damage-associated molecular patterns (DAMPs), i.e. plant-derived molecules that are generated or released as a result of wounding [20,21]. A more specific second layer of defense may be activated by insect-derived effector molecules, so-called herbivore-associated molecular patterns (HAMPs) [21,22], including fatty acid-amino acid conjugates (FACs) [23,24], caeliferins [25], bruchins [26], and inceptins [27,28]. In addition to these low-molecular weight compounds, several proteins were shown to be active as elicitors of plant defense, including glucose oxidase (GOX) [29,30] and β-glucosidase [31]. HAMPs and other insect-derived elicitors are produced in different combinations and quantities by different insects [24,32,33], and the response they elicit depends on the plant species [34]. They are thus likely to account for much of the specificity observed in plant-herbivore interactions.

The activation of plant defense by non-specific (DAMPs) and specific cues (HAMPs) alike depends on the jasmonate pathway as the core signaling machinery [20,21,35-37]. Mechanical wounding is sufficient to trigger the rapid and transient accumulation of jasmonic acid (JA) concomitant with its bioactive isoleucine conjugate (JA-Ile) in damaged as well as in systemic leaves [20,38-40]. On top of the basal induction by wounding, the production of JA/JA-Ile is potentiated by HAMPs that are present in insect oral secretions [21,24,41]. JA-Ile then promotes the CORONATINE-INSENSITIVE 1 (COI1)-dependent ubiquitinylation and degradation of repressor proteins leading to the transcriptional activation of defense responses [42,43]. Well-known markers of the JA/JA-Ile-mediated defense response in tomato include proteinase inhibitors I and II (PI-I and PI-II) and polyphenol oxidase (PPO) which serve an anti-nutritive role by reducing the digestibility of dietary protein [44-46].

To achieve specificity in their response to different herbivores, plants may engage additional signals acting in parallel to the JA cascade, or else, use other hormones as spatio-temporal modulators of the JA/JA-Ile response [21]. Recent findings actually suggest that most if not all plant hormones participate in the fine tuning of defense responses [21,47-50], and the integration of defense and development [51-54]. The question of whether other members of the jasmonate family may also contribute to specificity in plant-insect interactions has received less attention. Such a role may be attributed to 12-oxophytodienoic acid (OPDA), a substrate of OPDA reductase 3 (OPR3) in the jasmonate biosynthetic pathway and precursor of JA/JA-Ile [55-58].

A role for OPDA as a defense regulator is supported by the Arabidopsis *opr3* mutant, which is unable to metabolize OPDA and fails to synthesize downstream jasmonates [57]. The *opr3* mutant retains resistance against *Bradysia impatiens* and *Alternaria brassicicola* [58,59] and partial resistance against *Sclerotinia sclerotiorum* [60], suggesting that JA/JA-Ile is dispensable as a defense signal and may be substituted by OPDA. OPDA was in fact shown to elicit the synthesis of diterpenoid-derived volatiles in lima bean and the accumulation of phytoalexins in soybean more efficiently than JA [61,62]. In Arabidopsis, defense genes were found to be induced by OPDA, showing only partial overlap with those regulated by JA/JA-Ile, and including COI1-dependent as well as COI1-independent genes [58,63-66].

**Figure 1:**
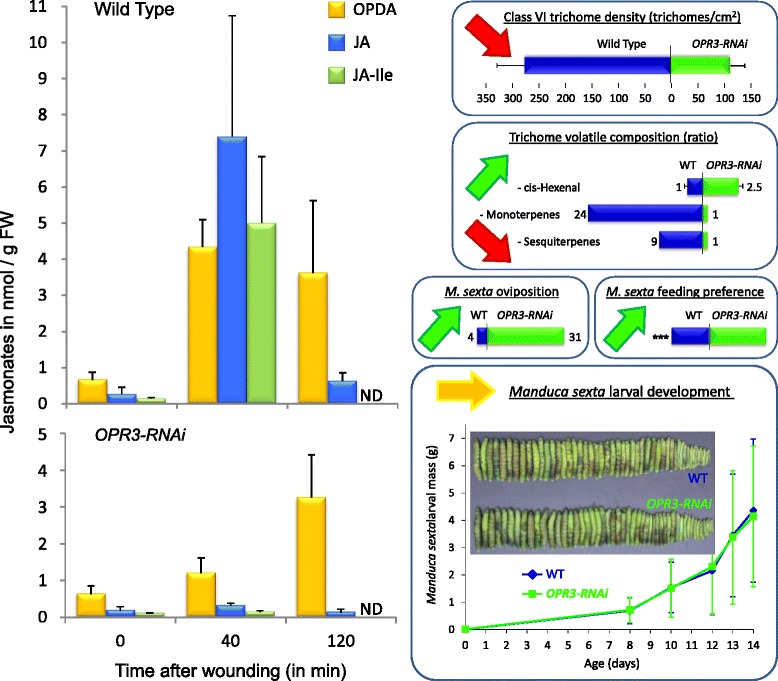
**Jasmonate levels and defense-related phenotypes of transgenic tomato plants silenced for** ***OPR3*** **expression by RNAi.** The figure summarizes the main findings of study [40] addressing the effect of JA/JA-Ile deficiency of *OPR3*-silenced plants on constitutive and induced defenses against the specialist herbivore *M. sexta* (green, red and yellow arrows indicating up- or down-regulation and no change in *OPR3-RNAi* as compared to control plants, respectively). *OPR3-RNAi* plants contain less JA/JA-Ile as compared to the wild type, and there is no wound-induced increase in JA or JA-Ile (left panel). As a result of JA/JA-Ile deficiency, trichome density and terpene content are reduced, while *cis*-3-hexenal concentration is increased in *OPR3-RNAi* as compared to wild-type plants (right panel, top). *OPR3-RNAi* plants are preferred by gravid *M. sexta* females for oviposition, and by the larvae for feeding (right panels, center). The development of *M. sexta* larvae is indistinguishable on *OPR3-RNAi* and wild-type plants (right panel, bottom). Resistance against larval feeding is thus maintained in the absence of JA/JA-Ile and was attributed to the local induction of defense gene expression by OPDA.

Using transgenic plants silenced for *OPR3* expression by RNA interference (RNAi) we recently showed that OPDA also acts as a defense signal in tomato [40]. Being impaired in the production of JA/JA-Ile from OPDA, *OPR3-RNAi* plants allowed us to assess the relative contributions of OPDA and JA/JA-Ile to constitutive and induced herbivore defense. We will refer to this study numerous times and, therefore, the main findings are summarized in Figure 1. *OPR3-RNAi* plants responded to wounding or OPDA treatment with the local induction of herbivore defense gene expression resulting in wild-type levels of resistance against tobacco hornworm (*Manduca sexta*) [40] (Figure 1). Constitutive defense traits, on the other hand, were compromised in *OPR3-RNAi* plants (Figure 1). This included a reduction in trichome density and terpene content leading to increased attraction of *M. sexta* moths for oviposition. The concentration of *cis*-3-hexenal, on the other hand, was found to be higher in *OPR3*-silenced as compared to control plants. *Cis*-3-hexenal acted as a feeding stimulant for *M. sexta* larvae resulting in increased leaf palatability and a preference for the JA/JA-Ile deficient over the wild-type genotype in dual-choice tests [40] (Figure 1). In the present study we included a second insect, *Spodoptera exigua* (beet armyworm), to compare the impact of JA/JA-Ile and OPDA-controlled defense traits on the resistance of tomato plants against specialist (*M. sexta*) and generalist (*S. exigua*) herbivores from the same feeding guild.

As previously shown for *M. sexta*, we found that *S. exigua* preferred JA/JA-Ile-deficient *OPR3-RNAi* tomato over wild-type plants for both oviposition and feeding. The behavior of both insects is thus controlled in a JA/JA-Ile-dependent manner, but different chemical cues were found to be responsible in the two species. In contrast, induced defense responses of tomato plants against *S. exigua* and *M. sexta* caterpillars differed with respect to their requirement of OPDA and JA/JA-Ile. While the OPDA-mediated induction of local defense is sufficient to restrict growth and development of *M. sexta* larvae, resistance against *S. exigua* was found to depend on additional defense traits, including the JA/JA-Ile dependent induction of foliar polyphenol oxidase (PPO) activity in response to *S. exigua* oral secretions.

## Results

### Jasmonate-dependent defense traits control oviposition and feeding behavior of *S. exigua*

**Figure 2:**
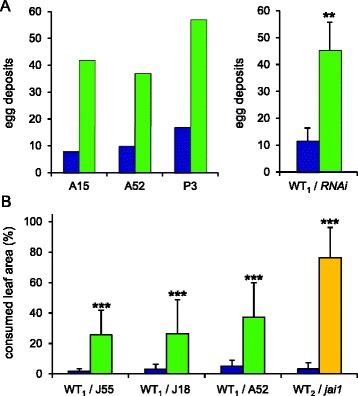
***S. exigua*** **prefers JA-deficient over wild-type plants for oviposition and feeding. (A)** Oviposition preference was analyzed in dual-choice assays as the number of egg deposits on *OPR3-RNAi* (green bars) as compared to wild-type plants (WT_1_, UC82B; blue bars). Data are shown for three independent *OPR3-RNAi* lines individually on the left (lines A15, A52, and P3), and as the mean of the three lines +/− SD on the right (paired t-test: **P = 0.007). **(B)** Feeding preference was analyzed in dual-choice assays using three independent *OPR3-RNAi* lines (green) and the *jai1* mutant (yellow) with the corresponding wild types (WT_1_, UC82B; WT_2_, Castlemart; blue). Each experiment consisted of three mutant and wild-type leaf discs offered to three larvae for feeding. Preference is shown as percent consumed leaf area after four hours. Data represent the mean +/− SD of at least 20 replicates (n = 28, 27, 37, and 20 for J55, J18, A52, and the *jai1* mutant). Asterisks indicate significant preference (Wilcoxon signed rank test: ***P < 0.001).

To assess the impact of JA/JA-Ile deficiency on host plant selection for oviposition by *S. exigua*, three male and female moths were caged in an insect tent with wild-type and *OPR3-RNAi* plants, two of each genotype. The plants were changed daily until oviposition was completed, and the number of egg deposits on each of the two genotypes was counted. Like previously shown for *M. sexta* [40], *S. exigua* moths showed a clear preference for JA/JA-Ile deficient plants, with 136 egg deposits on *OPR3-RNAi* as compared to 35 on wild-type plants (Figure 2A). *OPR3-RNAi* plants thus appear to lack defense trait(s) that deter both the generalist and the specialist herbivore from oviposition.

We then used JA/JA-Ile-deficient *OPR3-RNAi* plants and the JA/JA-Ile insensitive *jai1* mutant [67] to assess the impact of jasmonate biosynthesis and signaling on the feeding behavior of *S. exigua* larvae. In dual-choice tests, three leaf discs of either the *OPR3-RNAi* or the *jai1* mutant and the corresponding wild type (UC82B and Castlemart, respectively) were arranged alternately at the rim of a petri dish, and three fifth-instar *S. exigua* larvae were placed in the center. After four hours of feeding, the consumed leaf area was determined. A strong preference was observed for three independent *OPR3-RNAi* lines as well as the *jai1* mutant over the respective wild-type genotypes (Figure 2B).

**Figure 3:**
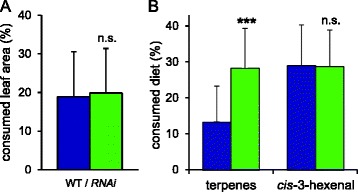
**Feeding preference of** ***S. exigua*** **larvae is determined by terpene content. (A)** Dual-choice test for feeding preference comparing trichome-cured wild-type (blue) and *OPR3-RNAi* leaves (green) were performed as in Figure 2B. The consumed leaf area is shown in percent as the mean +/− SD of 58 experiments. Differences between the means are not significant (Wilcoxon signed rank test: P = 0.895). **(B)** Dual-choice tests comparing artificial diet to which *cis*-3-hexenal (n = 44) or a blend of mono- and sesquiterpenes (n = 86) were added in concentrations reflecting the content of wild-type (blue) or *OPR3-RNAi* trichomes (green). Diet consumption after 20 hrs is shown in percent as the mean +/− SD. Asterisks indicate significant preference (Wilcoxon signed rank test: ***P < 0.001).

Since the JA/JA-Ile biosynthesis (*OPR3-RNAi*) and signaling (*jai1*) mutants are both impaired in trichome development and show a similar ~70% reduction in type VI glandular trichome density [40,67], we suspected that host plant choice of beet armyworm larvae may depend on trichome density and/or the levels of trichome-borne metabolites. Confirming a role for trichomes and their chemical constituents, any feeding preference was lost in dual-choice tests comparing *OPR3-RNAi* and wild-type plants from which leaf surface trichomes had previously been removed (Figure 3A).

We then tested the role of those trichome metabolites which were previously found to differ in concentration between *OPR3-RNAi* and wild-type plants: *cis*-3-hexanal with a 2.5-fold increase in *OPR3-RNAi* plants, and terpenes that are much reduced (monoterpenes: α-pinene (28-fold), 2-carene (18-fold), limonene (27-fold), α-phellandrene (22-fold), β-phellandrene (23-fold); sesquiterpenes: α-humulene (11-fold), δ-elemene (11-fold), β-caryophyllene (6-fold), Figure 1)[40]. The observed feeding preference of beet armyworm larvae may thus be caused either by a stimulating activity of *cis*-3-hexenal which is elevated in *OPR3-RNAi* plants, or else, by a reduction of repellent terpenes. To distinguish between these two possibilities, dual-choice tests were performed using artificial diet to which the synthetic compounds were added in amounts reflecting their concentrations in wild-type and *OPR3-RNAi* trichomes, respectively (Figure 3B).

In these experiments, *cis*-3-hexenal did not exert any effect on the feeding behavior of *S. exigua* (Wilcoxon signed rank test: P = 0.866), while a repellent activity was observed for the higher terpene concentrations of wild-type plants (Wilcoxon signed rank test: P < 0.001; Figure 3B). These findings for *S. exigua* are in striking contrast to those reported for *M. sexta* larvae, which are unresponsive to terpenes but incited to feed by *cis*-3-hexenal [40].

### Defense against *S. exigua* larvae is compromised in *OPR3*-RNAi plants and *jai1* mutants

**Figure 4:**
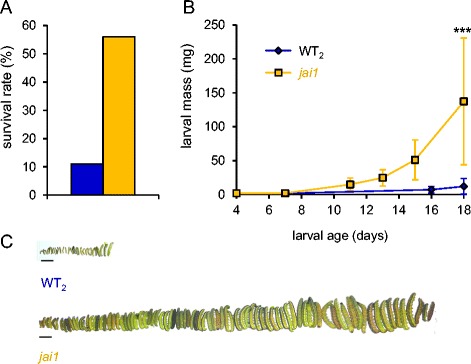
***S. exigua*** **larvae perform better on** ***jai1*** **mutants than on wild type.** The experiment involved 300 and 150 four-day-old larvae on wild-type and *jai1* plants, respectively. **(A)** Percent survival of *S. exigua* larvae on wild type (WT_2_, Castlemart, blue) and the *jai1* mutant (yellow). **(B)** Larval development on wild-type (blue) and *jai1* (yellow) host plants. Larval mass is given in mg as the mean +/− SD. Asterisks indicate significant differences (Wilcoxon signed rank test: *** P < 0.001). **(C)** *S. exigua* larvae at the end of the experiment, prior to pupation (scale bar = 1 cm).

**Figure 5:**
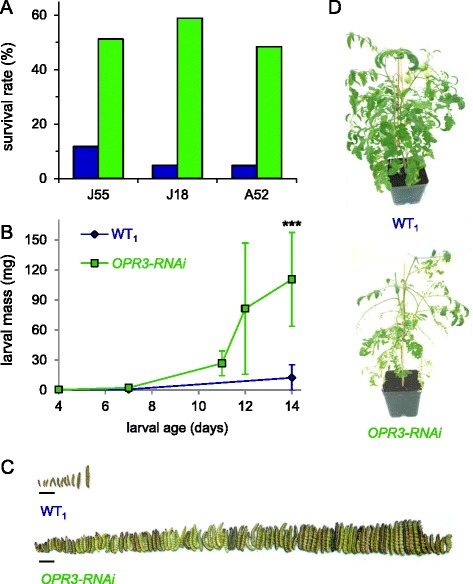
***S. exigua*** **larvae perform better on** ***OPR3-RNAi*** **plants than on wild type.** The experiment involved 246 and 175 four-day-old larvae on wild-type and *OPR3-RNAi* plants, respectively. **(A)** Percent survival of *S. exigua* larvae on wild type (WT_1_, UC82B, blue) and 3 independent *OPR3-RNAi* lines (J55, J18, A52; green). **(B)** Larval development on wild-type (blue) and *OPR3-RNAi* (green) host plants. Larval mass is given in mg as the mean +/− SD. Asterisks indicate significant differences (Wilcoxon signed rank test: *** P < 0.001). **(C)** *S. exigua* larvae at the end of the experiment, prior to pupation (scale bar = 1 cm). **(D)** One representative of wild-type and *OPR3-RNAi* host plants at the end of the experiment.

Wild-type tomato plants turned out to be a rather poor host for *S. exigua* with only 5–12% of the larvae surviving on the cultivars Castlemart (Figure 4A) and UC82B (Figure 5A). Mortality was much lower on *jai1* host plants, on which 56% of the larvae completed their development within 18 days reaching a weight of 137 mg just prior to pupation. At this time, the average weight of those that survived on wild type was only 12 mg (Figure 4B,C). These results are consistent with the central role of jasmonates in herbivore defense [8,21,37] and they further indicate that JAI1-dependent signaling and defense gene regulation are required for resistance against *S. exigua* larvae (Figure 4B,C) as previously shown for *M. sexta* [40].

On *OPR3-RNAi* host plants, growth and development of *S. exigua* was comparable to *jai1* (Figure 5). The rate of survival was about 50% on three independent transgenic lines (Figure 4A). Development was completed after 14 days when larvae weighed 110 mg as compared to 12 mg on wild type (Figure 5B,C) suggesting a loss of resistance against beet armyworm in *OPR3-RNAi* as compared to wild-type plants (Figure 5D). In contrast, resistance against tobacco hornworm is not compromised in *OPR3-RNAi* plants; despite the lack of JA/JA-Ile, *OPR3-RNAi* plants restricted *M. sexta* growth and development to the same extent as the wild type [40] (Figure 1).

We conclude that the defense traits that are active in tomato plants against *M. sexta* and *S. exigua* are not the same. While both depend on JAI1, they differ with respect to their requirement of JA/JA-Ile synthesis. Since defense against *M. sexta* is operating in *OPR3-RNAi* plants, conversion of OPDA to JA/JA-Ile is not required. Defense against *S. exigua*, on the other hand, is lost in *OPR3-RNAi* plants and, therefore, relies on (additional) traits that depend on JA/JA-Ile formation.

**Figure 6:**
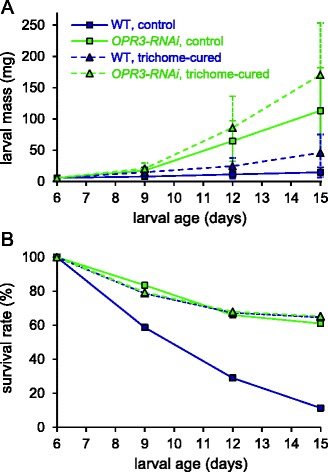
**Performance of** ***S. exigua*** **larvae on trichome-cured** ***OPR3-RNAi*** **and wild-type plants (broken lines) as compared to untreated controls (solid lines). (A)** Larval development on wild-type (blue) and *OPR3-RNAi* (green) host plants. Larval mass is given in mg as the mean +/− SD. **(B)** Percent survival of *S. exigua* larvae on wild-type (blue) and *OPR3-RNAi* (green) host plants. 300 and 150 larvae were used on untreated and trichome-cured wild type, while 200 and 150 larvae were used on untreated and trichome-cured *OPR3-RNAi* plants, respectively.

Among the defense traits that are compromised by JA/JA-Ile deficiency in *OPR3-RNAi* plants and shown here to contribute to their increased attractiveness to *S. exigua* are type VI glandular trichomes and their terpene constituents (Figure 3A,B). We therefore tested whether the differences in larval growth and development may be due to differences in host plant trichome density. The development of *S. exigua* larvae was analyzed on *OPR3-RNAi* and wild-type plants from which trichomes had previously been removed, and compared to the untreated controls. Larval development was marginally improved on both trichome-cured genotypes, but the large difference in their suitability as a host for *S. exigua* larvae was maintained: after 15 days, when beet armyworm larvae had completed development on trichome-cured *OPR3-RNAi* plants, they averaged 170 mg in weight as compared to 46 mg on the trichome-cured wild type (Figure 6A). In conclusion, growth and development of *S. exigua* must be restricted on wild-type as compared to *OPR3-RNAi* plants by JA/JA-Ile-dependent defense trait(s) other than trichome density and composition.

While the presence or absence of trichomes could not explain the observed difference in growth of beet armyworm larvae, it did have a major impact on their mortality: Only 11% of the larvae survived after 15 days on wild-type plants as compared to 61% on *OPR3-RNAi* (Figure 6B). On the trichome-cured genotypes, on the other hand, the rate of survival was indistinguishable at 65% (Figure 6B). Reduced mortality on trichome-cured plants is likely due to the removal of toxic terpenes [68].

**Figure 7:**
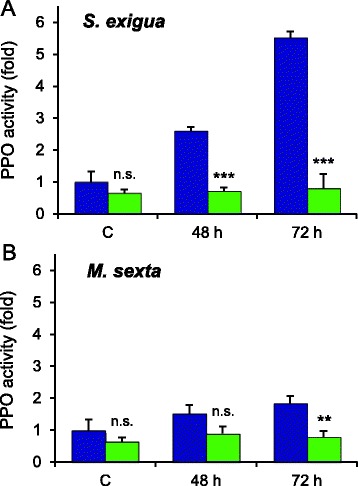
**Induction of PPO activity by** ***S. exigua*** **and** ***M. sexta*** **feeding.** PPO activity was assayed in wild-type (blue) and *OPR3-RNAi* plants (green) before (C), 48 and 72 hours after insect feeding. **(A)** PPO induction by *S. exigua*. **(B)** PPO induction by *M. sexta*. Data were normalized to PPO levels in unwounded wild-type controls and represent the mean +/− SD of 2 to 3 independent experiments each with four leaf samples. Significant differences between wild-type and *OPR3-RNAi* plants are indicated (t-test; ** P < 0.01, *** P < 0.001).

### JA/JA-Ile-dependent induction of polyphenol oxidase activity

Since polyphenol oxidase (PPO) is known to be part of the jasmonate-dependent inducible defense system against Lepidopteran insects [45], we tested whether differences in PPO activity can account for the observed differences in larval performance on *OPR3-RNAi* and wild-type plants. In healthy *OPR3-RNAi* plants, PPO activity appeared to be somewhat lower than in wild-type plants, but the difference was not statistically significant (Figure 7). In response to beet armyworm feeding, a strong induction of PPO activity was observed after 48 and 72 hours in wild-type plants (Figure 7A). There was no increase in activity in *OPR3-RNAi* plants indicating that JA/JA-Ile formation is required and that the JA precursor OPDA cannot substitute for JA/JA-Ile as a signal for PPO induction. Interestingly, the induction of PPO activity after *M. sexta* herbivory was much attenuated (Figure 7B) as compared to *S. exigua* feeding (Figure 7A). This observation suggests that the induction of PPO activity is not caused by wounding alone, but rather depends on the specific plant-insect interaction. Tomato plants obviously respond differently to *S. exigua* and to *M. sexta* feeding suggesting that insect-derived molecules in oral secretions are likely to be responsible for the observed differences in PPO induction.

**Figure 8:**
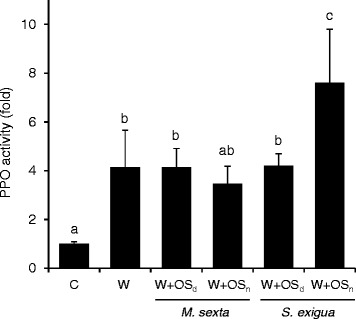
**Induction of PPO activity by mechanical wounding and insect oral secretions.** PPO activity was assayed in wild-type leaves 72 hours after mechanical wounding (W) or wounding with addition of insect (*M. sexta* or *S. exigua*) oral secretions (W + OS). OS were diluted 1:1 in water and applied in their native state (OS_n_) or after heat denaturation (OS_d_). Data are shown for one of three independent experiments, representing the mean +/− SD of four biological replicates each including pooled leaf material from three plants. Different letters indicate significant differences in PPO fold-induction normalized to unwounded controls (C; One-Way-ANOVA (F_5,18_ = 12.534, P < 0.001) and post-hoc Holm-Sidak for multiple comparisons at P < 0.05).

Therefore, we compared PPO induction in tomato leaves after mechanical wounding with the induction caused by wounding and the additional treatment with oral secretions (OS) of *M. sexta* or *S. exigua* (Figure 8). Mechanical wounding resulted in a modest induction of foliar PPO activity. There was no difference in PPO induction when native OS (OS_n_) of *M. sexta* were applied into the wound site. The application of native *S. exigua* OS, on the other hand, caused a substantial increase in PPO activity (Figure 8). These observations suggest that *M. sexta* is not actively suppressing wound-induced PPO activity but may rather be lacking an elicitor that is present in OS only of *S. exigua*. To find out to which class of molecules this putative elicitor may belong, we performed the same experiments with OS that had been denatured by heat treatment (OS_d_). Interestingly, after heat-treatment, the PPO inducing activity of *S. exigua* OS was no longer different from wounding or *M. sexta* OS (Figure 8). The induction of PPO activity is thus mediated by a heat-labile, likely proteinaceous constituent that is present in *S. exigua* but not in *M. sexta* OS. FACs that are known to differ in composition in the OS of the two insect species [33] are heat-stable [69] and thus unlikely to be responsible for the observed difference in the elicitation of plant defense.

## Discussion

In this study, we analyzed the impact of jasmonate-dependent defense traits of tomato plants on the generalist herbivore *S. exigua* and compared it to previous findings for the specialist *M. sexta*. To assess the relevance of the jasmonate precursor OPDA and JA/JA-Ile as signaling molecules for constitutive and induced plant defense against these insects, we used transgenic plants impaired in the conversion of OPDA to JA and JA-Ile (*OPR3-RNAi* plants) [40] and the JA/JA-Ile insensitive *jai1* signaling mutant [67].

### Feeding preference for JA/JA-Ile deficient plants is caused by different chemical cues for *S. exigua* and *M. sexta*

A reduction in trichome density and trichome-borne metabolites were previously shown to render *OPR3-RNAi* plants more attractive to *M. sexta* with respect to feeding and oviposition [40] (Figure 1). The altered oviposition behavior was attributed to reduced concentrations of repellent mono- and sesquiterpenes, whereas feeding preference was caused by an increase in *cis*-3-hexenal serving as a feeding stimulant for *M. sexta* larvae [40]. In the present study, we observed a similar preference of *S. exigua* for JA/JA-Ile deficient *OPR3-RNAi* plants during oviposition and feeding (Figure 2). However, unlike *M. sexta*, *S. exigua* larvae were impartial to the presence or absence of *cis*-3-hexenal. Feeding behavior was rather determined by differences in terpene content (Figure 3). Different chemical cues are thus perceived by *S. exigua* and *M. sexta*, resulting in similar behavioral responses in the two species.

### OPDA is insufficient as a signal for induced defense against *S. exigua*

In contrast to the constitutive defense traits that were impaired in *OPR3-RNAi* plants as well as in *jai1* mutants, some aspects of induced defense were unaffected by the silencing of OPR3. The induction of defensive proteinase inhibitor (*PI-II*) expression was observed in wounded leaves of *OPR3-RNAi* plants indicating that this process does not rely on the formation of JA/JA-Ile. OPDA was identified as a signal for *PI-II* expression that is sufficient for the local response in injured leaves but unable to substitute for JA/JA-Ile in the systemic wound response [40]. These findings added to the growing body of evidence for OPDA being a bioactive jasmonate differing in activity from JA/JA-Ile [58,63,70-72].

Interestingly, there was a pronounced difference in performance of *S. exigua* and *M. sexta* larvae on the JA/JA-Ile biosynthesis and signaling mutants suggesting that the impact of OPDA- and JA/JA-Ile-mediated defenses differs in these two species. *S. exigua* larval development and weight gain were similar on the JA/JA-Ile-deficient and on JA/JA-Ile-insensitive genotypes. In both cases larvae consumed much more leaf material, gained weight more rapidly and developed faster as compared to those reared on wild-type host plants (Figures 4 and 5). Consistent with this observation, Thaler et al. reported reduced mortality of *S. exigua* on the JA-deficient *def1* tomato mutant [73]. These findings indicate that JA/JA-Ile formation and signaling are both required for resistance against *S. exigua*. In contrast, performance of *M. sexta* larvae was improved only on the *jai1* mutant, not on *OPR3-RNAi* plants. OPDA-mediated induction of defensive proteinase inhibitors thus appears sufficient to confer resistance against *M. sexta*, but not against *S. exigua*. Consistent with this observation, Jongsma et al. reported that growth of *S. exigua* larvae is unaffected by high levels of potato PI-II in their diet [74]. The larvae compensate for the loss of digestive activity by induction of proteases that are insensitive to PI-II inhibition [74]. These findings imply the existence of additional defense trait(s) in tomato for induced resistance against *S. exigua* and the induction of these traits appears to depend on JA/JA-Ile.

### JA/JA-Ile-dependent induction of foliar PPO activity limits performance of *S. exigua*

Polyphenol oxidase (PPO) is a reliable marker for JA-induced defense in tomato and a good predictor of insect performance [45,73,75,76]. PPOs oxidize plant phenolics to highly reactive quinones that form Michael adducts with cellular nucleophiles, including DNA, lipids, proteins and amino acids. In the insect gut, PPOs reduce the nutritive quality and digestibility of dietary proteins and the availability of essential amino acids [46,77,78]. In addition to this post-ingestive activity, limited oxygen availability in the insect gut argues for a further pre-ingestive function of PPO [79]. Supporting a role in plant defense, resistance to herbivory is enhanced in transgenic hybrid aspen overexpressing PPO [80], and relative growth rate of *M. sexta* is negatively correlated with PPO activity in different tomato tissues [81]. A defensive role of PPO has also been demonstrated against *S. exigua* using artificial diet [78], defense-signaling mutants in Arabidopsis [82], and transgenic tomato plants with altered PPO expression in an otherwise identical genetic background [77], These findings prompted us to test whether differences in PPO activity can account for the observed differences in the performance of *S. exigua* larvae on *OPR3-RNAi* and wild-type plants. Consistent with this hypothesis, we found PPO activity to be induced in response to *S. exigua* feeding in a JA/JA-Ile-dependent manner in wild-type tomato but not in *OPR3-RNAi* plants (Figure 7A), and this lack of PPO induction in *OPR3-RNAi* plants correlated with a loss of resistance and improved larval development (Figure 5).

### Differential induction of PPO activity by *S. exigua* and *M. sexta*

Interestingly, the induction of PPO activity in response to *M. sexta* feeding was much lower as compared to *S. exigua* (Figure 7). Consistent with the lower level of induced PPO in response to *M. sexta*, tomato is a much better host for *M. sexta* than *S. exigua*. Species-specific differences in plant responses to herbivory are a likely result of co-evolution. In the co-evolutionary arms race, many insects acquired the ability to manipulate plant defense, and this ability is expected to differ with the degree of host specialization [14]. According to this hypothesis, generalist herbivores are predicted to have evolved ‘general’ mechanisms to tolerate an array of plant defenses, and to possess the tools to manipulate their host plants by interfering with highly conserved defense signaling pathways [14]. Many generalists were in fact shown to exploit the antagonism between the SA and JA signaling pathways to attenuate JA-mediated defense responses [33,82-87]. Similarly, Colorado potato beetle larvae were shown to exploit the conserved non-host resistance response triggered by microbe-associated molecular patterns to counteract host defenses in tomato [86]. While the interaction of specialists with their host plants may involve additional more specific signals and more restricted signaling pathways, this can also result in a down-regulation of host defenses. The spider mite *Tetranychus evansi*, for example, is able to minimize the induction of direct (proteinase inhibitor accumulation) and indirect (volatile emission) defenses in tomato plants [88]. Oral secretions of Colorado potato beetle were found to suppress the wound-induced expression of defense genes in tomato [89,90] and potato [91]. Likewise, oral secretions of *M. sexta* antagonize induced nicotine production in *N. attenuata* [92,93], and *Ectropis obliqua*, a major insect pest of tea, uses OS to elude its host plant’s defense by inhibiting the production of PPOs [94]. In contrast, we did not observe any effect of adding *M. sexta* OS on the level of PPO induction as compared to wounding alone (Figure 8). It was further shown by others that tomato plants are unresponsive to three classes of elicitors (FACs, inceptin, caeliferin) from OS of different insects [34]. The active suppression of PPO activity by *M. sexta* OS is thus unlikely.

A change in perspective offers an alternative explanation for the differential induction of plant defense responses by generalist and specialist herbivores. Rather than being beneficial for the insect, attenuation of defense responses after specialist attack could also be an adaptation of the host. Looking at the interaction from the plant’s point of view, low-levels of induced defense may be beneficial if the attacker is able to use host defenses to its own advantage [14]. Reduced production of toxic secondary metabolites, for example, may provide an advantage against specialists that co-opt diet-derived toxins for their own defense [95,96]. Accordingly, the suppression of nicotine production by OS from *M. sexta* has been interpreted as an adaptive response of the host [92,93]. *N. attenuata* plants challenged by *M. sexta* in their native habitat do indeed benefit from low nicotine content, as larvae raised on nicotine-free host plants suffer higher rates of predation by wolf spiders [97]. However, since PPO-based defense is also operating against *M. sexta*, with larval growth rates being negatively correlated to leaf PPO activity in tomato [81], it is hard to see how the plant could benefit from low PPO induction. Therefore, comparing the strong induction of PPO activity by *S. exigua* OS to the low induction by *M. sexta* OS and wounding (Figure 8), the most likely explanation for the differential induction is the presence of an elicitor in *S. exigua* OS that is missing in OS from *M. sexta*.

### Heat-labile elicitor of PPO activity and defense against *S. exigua*

The difference in PPO-inducing activity between OS from *S. exigua* and *M. sexta* was lost after heat-treatment (Figure 8), suggesting that the putative elicitor is a protein, possibly an enzyme, rather than FACs which were shown to be heat-stable [69,98]. An obvious candidate is glucose oxidase (GOX). GOX was first identified as a suppressor of plant defense in labial saliva of *Helicoverpa zea,* inhibiting nicotine production in tobacco [30]. Also in *N. attenuata*, GOX interferes with hormone-signaling, down-regulating JA/JA-Ile dependent defense responses against *S. exigua* [33]. However, GOX may also act as an elicitor of plant defense: The induction of foliar PPO activity in tobacco was stronger in response to the generalist *H. armigera*, and this correlated with 10-fold higher GOX activity in labial glands of *H. armigera* as compared to the specialist *H. assulta* [99]. Similarly, we observed stronger induction of PPO activity in tomato leaves after generalist (*S. exigua*) than specialist (*M. sexta*) feeding (Figure 7). The level of PPO induction correlates with GOX activity that was reported by others to be higher in *S. exigua* as compared to *M. sexta* OS [33]. These observations support GOX as a possible elicitor of defensive PPO in tomato, implying that tomato plants may be able to distinguish between attack by *S. exigua or M. sexta* on basis of different GOX levels in insect OS.

Our findings are consistent with data from the Felton lab, showing that induction levels of defensive proteinase inhibitors in tomato correlate with GOX activity in salivary gland homogenates from different species, being highest for *S. exigua* and lowest for *Trichoplusia ni* and *M. sexta* [100]. Since GOX is part of the herbivore’s offensive effector repertoire suppressing plant defense in most species, the specific recognition and elicitation of defense in tomato has been likened to effector-triggered immunity in plant pathogen interactions [100]. Effector-triggered immunity results from the specific resistance (R) gene-dependent detection of a pathogen effector by the host’s surveillance system [101]. Pathogens lacking the effector protein escape detection resulting in a compatible interaction and the development of disease. It may thus be envisaged that rather than being lost in the course of co-evolution, the low level of GOX in OS may have been a critical factor facilitating the initial colonization of Solanaceous host plants by *M. sexta*.

## Conclusions

Using mutants and transgenic plants affected in JA/JA-Ile biosynthesis or signaling, we analyzed the relevance of OPDA- and JA/JA-Ile-dependent traits of tomato plants for resistance against two insects, the generalist *S. exigua* and the specialist *M. sexta*. Both insects preferred JA/JA-Ile deficient plants for oviposition and feeding. Feeding preference for JA/JA-Ile-deficient plants was found to be caused by different chemical cues in the two species, the lack of repellant mono- and sesquiterpenes for *S. exigua*, and increased levels of *cis*-3-hexenal acting as a feeding stimulant for *M. sexta*. Larval performance was differentially affected in plants impaired in JA/JA-Ile biosynthesis and signaling. The local induction of defense genes mediated by the JA/JA-Ile precursor OPDA was found to be sufficient to restrict growth and development of *M. sexta* larvae. Defense against *S. exigua*, on the other hand, relied on additional JA/JA-Ile dependent factors, including the induction of foliar PPO. A heat-labile constituent of larval OS was found to be responsible for the specific differences in defense responses of tomato plants against *S. exigua* and *M. sexta*.

## Methods

### Experimental plants

The generation and propagation of transgenic tomato plants silenced for the expression of *OPR3* (*OPR3-RNAi plants*) has been described [40]. All experimental plants were grown from T1 seeds and the presence of the sense and anti-sense parts of the silencing construct were confirmed by PCR (all PCR primers were obtained from Operon (Cologne, Germany) sense part: 5′-ATGCCTGATGGAACTCATGGGA-3′ and 5′-AGCGGAGAAATTCACAGAGCAGGA-3′; anti-sense part: 5′-ATGCCTGATGGAACTCATGGGA-3′ and 5′-TGTGGCAATCCCTTTCACAACCTG-3′). Silencing was confirmed by western blot analysis using a polyclonal antiserum directed against recombinant OPR3 expressed in *E. coli* [40]. Tomato UC82B (Royal Sluis, The Netherlands) was used as the corresponding wild-type control in all experiments involving *OPR3-RNAi* plants.

Gregg Howe (Michigan State University, East Lansing, USA) kindly provided segregating F2 seeds of the *jai1* mutant in the Castlemart background [67]. Homozygous mutants were identified by PCR. Specific primer pairs were used to distinguish the wild-type (JAI-1-F: 5′-GTGGAGACGATATGTTGAGACTAA-3′ and JAI-1-R: 5′-CCATGGAGTCCATCACCTAACAGT-3′) and the mutant allele (JAI-1-F and *jai*-1-R: 5′-GTGGTCAGATCAGAGCCCTCTATT-3′), yielding amplicons of 525 bp and 777 bp, respectively.

To minimize the risk of Tobamovirus infections from potentially contaminated seeds, they were incubated over night at 70°C, sterilized in 70% ethanol for 5 min, rinsed in water, incubated in 10% (w/v) trisodium phosphate for 3 hours, and finally rinsed in 5 changes of water for 5 min each prior to sowing. Plants were cultivated in the greenhouse with supplemental light at 16 hours photoperiod and 26°C/18°C day/night temperature regime. Experimental plants were fertilized at weekly intervals and excluded from phytosanitary procedures.

### Insect culture

*Spodoptera exigua*, Hübner (Lepidoptera: Noctuidae) eggs were kindly provided by Michael Rostás (University Würzburg, Germany) and Sascha Eilmus at Bayer CropScience AG (Monheim, Germany). Eggs were surface sterilized by exposure to gaseous formaldehyde (4–16 hrs, 5% v/v) and ammonia (20 min, 0.625% v/v) followed by 30 min aeration. Larvae were raised in plastic boxes (20 x 10 x 6 cm for early instars, 20 x 20 x 5 cm for late instars) at 22°C on artificial noctuid diet (500 μl corn oil, 580 mg Wesson’s salt mix (Sigma-Aldrich; Steinheim, Germany), 3 g sorbic acid, 375 mg ethanolic methyl 4-hydroxybenzoate, 3.3 g ascorbic acid, 3.08 g Ain Vitamins (MP Biomedicals; Heidelberg, Germany), 33 g brewer’s yeast flakes, 15 g alfalfa leaf powder, 111 g bean flower, 500 mg sitosterol (50%, Applichem; Darmstadt, Germany), 417 mg *L*-leucine and 2 ml formaldehyde (37%) added to 760 ml of autoclaved water with 18 g agar). Pupae were surface-sterilized for 10 min in 0.25% (v/v) sodium hypochlorite. For hatching, mating and oviposition, 5 males and females were joined in plexiglas cylinders (25 cm x 9,5 cm) lined with filter paper. 20% (w/v) sucrose on cotton wool was given as a food source. Oral secretions were collected from fifth-instar larvae as described [98]. The larvae had been raised on artificial diet and allowed to feed on wild-type tomato plants for 48 hours before collection.

*Manduca sexta*, Johanson (Lepidoptera: Sphingidae) eggs were kindly provided by Ian Baldwin, Danny Kessler and Celia Diezel (Max Planck Institute for Chemical Ecology, Jena, Germany). Eggs were surface-sterilized for 15 min in 10% formaldehyde and rinsed in a large volume of water. Eggs were incubated and larvae raised at 25°C and 16 hour photoperiod on gypsy moth wheat germ diet (MP Biomedicals), first in petri dishes and at later instars in 32 x 22 x 5 cm transparent plastic boxes. Just prior to pupation, larvae were wrapped in paper towels and stored at 25°C in the dark. Towards the end of pupal stage, they were unwrapped, covered in 5–10 cm perlite and transferred into BugDorm-2 insect tents (MegaView Science; Taichung, Taiwan) in a green house for hatching, mating, and finally oviposition on four- to six-week-old tomato plants. For collection of *M. sexta* oral secretions, conditions were the same as described above for *S. exigua*.

### Bioassays for feeding and oviposition preference

Feeding preference was analyzed in dual-choice tests using three leaf discs (Ø 2 cm) of two genotypes (*OPR3-RNAi* vs. UC82B, or *jai1* vs. Castlemart) that were placed alternately in a circle on top of a net covering a 9 cm water-filled petri dish. Three fifth-instar *S. exigua* larvae starved for two hours were placed in the center. The setup was covered with a glass beaker to maintain high humidity and prevent wilting of the leaves. After four hours, the feeding experiment was terminated and the consumed leaf area determined. The number of replicates was n = 28, 27, and 37 for three independent *OPR3-RNAi* lines and n = 20 for the *jai1* mutant. To assess the impact of trichomes and their chemical constituents on feeding behavior, the same experiments was performed with leaves that had been wiped with methanol-saturated cotton wool to remove trichomes, and then rinsed in an excess of water (n = 58).

The effect of terpenes and *cis*-3-hexenal on feeding preference was analyzed in dual-choice tests with artificial diet to which the test compounds were added in amounts reflecting the concentration in trichome extracts from wild-type or *OPR3-RNAi* plants. Six discs of artificial diet (1.2 cm in diameter, 2 mm thick, approx. 200 mg) were placed in two rows of three on opposing sides of a covered 11 cm x 7.5 cm plastic dish. A blend of commercially available terpenes (40 μl, in hexane) corresponding to the terpene content of 0.8 g of leaf tissue of either wild-type or *OPR3-RNAi* plants [40] was applied to each leaf disc at the beginning and again after four and 16 hours of the experiment (n = 86). In the same way, *cis*-3-hexenal was applied in 40 μl 50% (v/v) triacetyl glycerol in the concentration reflecting the hexenal content of wild-type and *OPR3-RNAi* plants, respectively (n = 44). Three fifth-instar *S. exigua l*arvae were placed in the center and allowed to feed for 20 hours. The consumed mass was determined as the weight difference before and after feeding, corrected for the weight loss by evaporation.

Oviposition preference was analyzed in dual-choice tests using BugDorm-2 insect tents, each containing two wild-type and two *OPR3-RNAi* plants. Three male and female moths were allowed to mate and make their choice for oviposition. Experimental plants were exchanged daily until oviposition was completed. Oviposition choice was quantified as the number of egg deposits (not the number of individual eggs) on each of the two genotypes. The experiment was performed with three independent *OPR3-RNAi* lines involving 15 replicates for lines A15 and A52, and 17 replicates for line P3.

### Bioassays for insect performance

Eight-week old *OPR3-RNAi* and wild-type (UC82B) plants (about 50 cm in height) were used to analyze insect performance and to compare host plant resistance. Four-day old *S. exigua* larvae were distributed on each of the two genotypes. To compensate for the difference in mortality, a larger number (246) were placed on wild-type as compared to *OPR3-RNAi* plants (175). Similarly, in the experiment comparing the *jai1* mutant to its corresponding wild type (Castlemart), 300 and 160 larvae were used, respectively. Laval weight was determined as indicated in Figures 4 and 5 and the experiment was terminated when larvae were ready to pupate.

To assess the impact of trichomes on insect performance, similar experiments were performed comparing larval development on wild type with trichome-cured wild type, and *OPR3-RNAi* with trichome-cured *OPR3-RNAi* plants. To remove trichomes, leaves were carefully wiped with methanol-soaked cotton wool and rinsed with water to remove residual methanol as described [68]. 300 and 150 larvae were used on untreated and trichome-cured wild type, while 200 and 150 larvae were used on untreated and trichome-cured *OPR3-RNAi* plants, respectively. Weight gain of the larvae was assessed at the time-points indicated in Figure 6 and the experiment was terminated when larvae on trichome-free *OPR3-RNAi* plants were ready to pupate.

### Polyphenol oxidase (PPO) activity assay

PPO activity was compared in unwounded healthy tomato leaves, in leaves that were wounded at 0, 24, and 48 hrs with a hemostat on two terminal leaflets, and in similarly wounded leaves with insect oral secretions added into the wound site (1:1 in water; 10 μl). Activity was assayed as described by Song et al. [102] with minor modifications. Leaf samples (approx. 0.5 g) were ground in liquid nitrogen, extracted in 1 ml 50 mM sodium phosphate buffer (pH 7,8), 1% (w/v) polyvinyl polypyrrolidone, 0.1 mM EDTA, and extracted for 1 hour at 4°C in an end-over-end shaker. Extracts were cleared by centrifugation (20 min at 16000 *xg*, 4°C) and protein concentration was determined according to Bradford [103] using bovine serum albumin as the reference protein. Protein extract (100 μg of protein in 150 μl) was added to 800 μl 50 mM (+)-catechin (Carl Roth; Karlsruhe, Germany) in 0.1 M sodium phosphate buffer pH 6.0. Assays were incubated for 15 min at 37°C and then quenched by addition of 150 μl 6 N HCl. OD_420_ was read against a reference that was quenched immediately. Specific PPO activity was calculated as units (U) per mg sample protein with 1 U corresponding to an absorbance change of 0.01/min. Since PPO activity levels of control plants were somewhat variable between independent experiments, data were normalized to the specific PPO activity in leaves of healthy wild-type controls and the results are shown as fold induction (with 1 corresponding to a specific activity of 3.2 to 23 U/mg).

### Statistics

SigmaPlot® 10.0 (Systat Software GmbH; Erkrath, Germany) was used for statistical analyses. In dual-choice tests for feeding and oviposition preference, differences in leaf consumption and egg deposition were analyzed using paired t-tests or the Wilcoxon signed rank test depending on whether data were normally distributed or not. Similarly, the unpaired t-test or Wilcoxon signed rank test was used to compare the means of larval weight gain and PPO induction of wild-type and mutant genotypes. Data on PPO induction by different wounding and elicitor treatments were analyzed by One-Way-ANOVA (F_5,18_ = 12.534, P < 0.001) followed by multiple pairwise comparisons of means using the post-hoc Holm-Sidak test. All statistical tests were performed at a threshold value of α = 0.05.

## Abbreviations

COI1: Coronatine-insensitive 1
DAMP: Damage-associated molecular patterns
FACs: Fatty acid-amino acid conjugates
GOX: Glucose oxidase
HAMP: Herbivore-associated molecular patterns
JA: Jasmonic acid
OPDA: 12-oxophytodienoic acid
OS: Oral secretions
PPO: Polyphenol oxidase
SA: Salicylic acid
R: Resistance
RNAi: RNA interference

## References

[1] Ehrlich PR, Raven PH (1964). Butterflies and plants: a study in coevolution. Ecology.

[2] Janz N (2011). Ehrlich and Raven revisited: Mechanisms underlying codiversification of plants and enemies. Ann Rev Ecol Evol Sys.

[3] Berlocher SH, Feder JL (2002). Sympatric speciation in phytophagous insects: moving beyond controversy?. Annu Rev Entomol.

[4] Cornell HV, Hawkins BA (2003). Herbivore responses to plant secondary compounds: A test of phytochemical coevolution theory. Am Nat.

[5] Walling LL (2000). The myriad plant responses to herbivores. J Plant Growth Regul.

[6] Züst T, Heichinger C, Grossniklaus U, Harrington R, Kliebenstein DJ, Turnbull LA (2012). Natural enemies drive geographic variation in plant defenses. Science.

[7] Howe GA, Schaller A, Schaller A (2008). Direct defenses in plants and their induction by wounding and insect herbivores. Induced plant resistance against herbivory.

[8] Howe GA, Jander G (2008). Plant immunity to insect herbivores. Ann Rev Plant Biol.

[9] Heil M (2008). Indirect defence via tritrophic interactions. New Phytol.

[10] Bruinsma M, Dicke M, Schaller A (2008). Herbivore-induced indirect defense: From induction mechanisms to community ecology. Induced plant resistance against herbivory.

[11] Clavijo McCormick A, Unsicker SB, Gershenzon J (2012). The specificity of herbivore-induced plant volatiles in attracting herbivore enemies. Trends Plant Sci.

[12] Wittstock U, Gershenzon J (2002). Constitutive plant toxins and their role in defense against herbivores and pathogens. Curr Opin Plant Biol.

[13] Dussourd DE, Eisner T (1987). Vein-cutting behavior: insect counterploy to the latex defense of plants. Science.

[14] Ali JG, Agrawal AA (2012). Specialist versus generalist insect herbivores and plant defense. Trends Plant Sci.

[15] Broekgaarden C, Voorrips RE, Dicke M, Vosman B (2011). Transcriptional responses of *Brassica nigra* to feeding by specialist insects of different feeding guilds. Insect Sci.

[16] Leitner M, Boland W, Mithofer A (2005). Direct and indirect defences induced by piercing-sucking and chewing herbivores in *Medicago truncatula*. New Phytol.

[17] Reymond P, Bodenhausen N, Van Poecke RM, Krishnamurthy V, Dicke M, Farmer EE (2004). A conserved transcript pattern in response to a specialist and a generalist herbivore. Plant Cell.

[18] Agrawal AA (2000). Specificity of induced resistance in wild radish: causes and consequences for two specialist and two generalist caterpillars. Oikos.

[19] Bidart-Bouzat MG, Kliebenstein D (2011). An ecological genomic approach challenging the paradigm of differential plant responses to specialist versus generalist insect herbivores. Oecologia.

[20] Heil M, Ibarra-Laclette E, Adame-Álvarez RM, Martínez O, Ramirez-Chávez E, Molina-Torres J, Herrera-Estrella L (2012). How plants sense wounds: Damaged-self recognition is based on plant-derived elicitors and induces octadecanoid signaling. PLoS ONE.

[21] Erb M, Meldau S, Howe GA (2012). Role of phytohormones in insect-specific plant reactions. Trends Plant Sci.

[22] Mithofer A, Boland W (2008). Recognition of herbivory-associated molecular patterns. Plant Physiol.

[23] Alborn HT, Turlings TCJ, Jones TH, Stenhagen G, Loughrin JH, Tumlinson JH (1997). An elicitor of plant volatiles from Beet Armyworm oral secretion. Science.

[24] Tumlinson JH, Engelberth J, Schaller A (2008). Fatty acid-derived signals that induce or regulate plant defenses against herbivory. Induced plant resistance to herbivory.

[25] Alborn HT, Hansen TV, Jones TH, Bennett DC, Tumlinson JH, Schmelz EA, Teal PEA (2007). Disulfooxy fatty acids from the American bird grasshopper *Schistocerca americana*, elicitors of plant volatiles. Proc Natl Acad Sci U S A.

[26] Doss RP, Oliver JE, Proebsting WM, Potter SW, Kuy S, Clement SL, Williamson RT, Carney JR, DeVilbiss ED (2000). Bruchins: Insect-derived plant regulators that stimulate neoplasm formation. Proc Natl Acad Sci U S A.

[27] Schmelz EA, Carroll MJ, LeClere S, Phipps SM, Meredith J, Chourey PS, Alborn HT, Teal PEA (2006). Fragments of ATP synthase mediate plant perception of insect attack. Proc Natl Acad Sci U S A.

[28] Schmelz EA, LeClere S, Carroll MJ, Alborn HT, Teal PEA (2007). Cowpea chloroplastic ATP synthase is the source of multiple plant defense elicitors during insect herbivory. Plant Physiol.

[29] Felton GW, Schaller A (2008). Caterpillar secretions and induced plant responses. Induced plant resistance to herbivory.

[30] Musser RO, Hum-Musser SM, Eichenseer H, Peiffer M, Ervin G, Murphy JB, Felton GW (2002). Herbivory: Caterpillar saliva beats plant defences. A new weapon emerges in the evolutionary arms race between plants and herbivores. Nature.

[31] Mattiacci L, Dicke M, Posthumus MA (1995). beta-Glucosidase: an elicitor of herbivore-induced plant odor that attracts host-searching parasitic wasps. Proc Natl Acad Sci U S A.

[32] Yoshinaga N, Alborn H, Nakanishi T, Suckling D, Nishida R, Tumlinson J, Mori N (2010). Fatty acid-amino acid conjugates diversification in Lepidopteran caterpillars. J Chem Ecol.

[33] Diezel C, von Dahl CC, Gaquerel E, Baldwin IT (2009). Different Lepidopteran elicitors account for cross-talk in herbivory-induced phytohormone signaling. Plant Physiol.

[34] Schmelz EA, Engelberth J, Alborn HT, Tumlinson JH, Teal PEA (2009). Phytohormone-based activity mapping of insect herbivore-produced elicitors. Proc Natl Acad Sci U S A.

[35] Heil M (2009). Damaged-self recognition in plant herbivore defence. Trends Plant Sci.

[36] Ballaré CL (2011). Jasmonate-induced defenses: a tale of intelligence, collaborators and rascals. Trends Plant Sci.

[37] Schaller A, Stintzi A, Schaller A (2008). Jasmonate biosynthesis and signaling for induced plant defense against herbivory. Induced plant resistance against herbivory.

[38] Glauser G, Grata E, Dubugnon L, Rudaz S, Farmer EE, Wolfender JL (2008). Spatial and temporal dynamics of jasmonate synthesis and accumulation in Arabidopsis in response to wounding. J Biol Chem.

[39] Koo AJK, Gao X, Jones AD, Howe GA (2009). A rapid wound signal activates the systemic synthesis of bioactive jasmonates in *Arabidopsis*. Plant J.

[40] Bosch M, Wright LP, Gershenzon J, Wasternack C, Hause B, Schaller A, Stintzi A (2014). Jasmonic acid and its precursor 12-oxophytodienoic acid control different aspects of constitutive and induced herbivore defenses in tomato. Plant Physiol.

[41] McCloud ES, Baldwin IT (1997). Herbivory and caterpillar regurgitants amplify the wound-induced increases in jasmonic acid but not nicotine in *Nicotiana sylvestris*. Planta.

[42] Browse J (2009). Jasmonate passes muster: a receptor and targets for the defense hormone. Ann Rev Plant Biol.

[43] Pauwels L, Goossens A (2011). The JAZ proteins: A crucial interface in the jasmonate signaling cascade. Plant Cell.

[44] Farmer EE, Ryan CA (1992). Octadecanoid precursors of jasmonic acid activate the synthesis of wound-inducible proteinase inhibitors. Plant Cell.

[45] Constabel CP, Bergey DR, Ryan CA (1995). Systemin activates synthesis of wound-inducible tomato leaf polyphenol oxidase via the octadecanoid defense signaling pathway. Proc Natl Acad Sci U S A.

[46] Felton GW (2005). Indigestion is a plant’s best defense. Proc Natl Acad Sci U S A.

[47] Robert-Seilaniantz A, Grant M, Jones JD (2011). Hormone crosstalk in plant disease and defense: more than just jasmonate-salicylate antagonism. Annu Rev Phytopathol.

[48] Verhage A, van Wees SC, Pieterse CM (2010). Plant immunity: it’s the hormones talking, but what do they say?. Plant Physiol.

[49] Hou X, Lee LYC, Xia K, Yan Y, Yu H (2010). DELLAs modulate jasmonate signaling via competitive binding to JAZs. Dev Cell.

[50] Hong G-J, Xue X-Y, Mao Y-B, Wang L-J, Chen X-Y (2012). Arabidopsis MYC2 interacts with DELLA proteins in regulating sesquiterpene synthase gene expression. Plant Cell.

[51] Meldau S, Erb M, Baldwin IT (2012). Defence on demand: Mechanisms behind optimal defence patterns. Ann Bot.

[52] Lan Z, Krosse S, Achard P, van Dam NM, Bede JC (2014). DELLA proteins modulate Arabidopsis defences induced in response to caterpillar herbivory. J Exp Bot.

[53] Qi T, Huang H, Wu D, Yan J, Qi Y, Song S, Xie D (2014). Arabidopsis DELLA and JAZ proteins bind the WD-repeat/bHLH/MYB complex to modulate gibberellin and jasmonate signaling synergy. Plant Cell.

[54] Heinrich M, Hettenhausen C, Lange T, Wünsche H, Fang J, Baldwin IT, Wu J (2013). High levels of jasmonic acid antagonize the biosynthesis of gibberellins and inhibit the growth of *Nicotiana attenuata* stems. Plant J.

[55] Schaller A, Stintzi A (2009). Enzymes in jasmonate biosynthesis - structure, function, regulation. Phytochemistry.

[56] Wasternack C, Hause B (2013). Jasmonates: biosynthesis, perception, signal transduction and action in plant stress response, growth and development. An update to the 2007 review in Annals of Botany. Ann Bot.

[57] Stintzi A, Browse J (2000). The *Arabidopsis* male-sterile mutant, *opr3*, lacks the 12-oxophytodienoic acid reductase required for jasmonate synthesis. Proc Natl Acad Sci U S A.

[58] Stintzi A, Weber H, Reymond P, Browse J, Farmer EE (2001). Plant defense in the absence of jasmonic acid: the role of cyclopentenones. Proc Natl Acad Sci U S A.

[59] Zhang Y, Turner JG (2008). Wound-induced endogenous jasmonates stunt plant growth by inhibiting mitosis. PLoS ONE.

[60] Stotz HU, Jikumaru Y, Shimada Y, Sasaki E, Stingl N, Mueller MJ, Kamiya Y (2011). Jasmonate-dependent and COI1-independent defense responses against *Sclerotinia sclerotiorum* in *Arabidopsis thaliana*: Auxin is part of COI1-independent defense signaling. Plant Cell Physiol.

[61] Koch T, Krumm T, Jung V, Engelberth J, Boland W (1999). Differential induction of plant volatile biosynthesis in the lima bean by early and late intermediates of the octadecanoid-signaling pathway. Plant Physiol.

[62] Fliegmann J, Schuler G, Boland W, Ebel J, Mithofer A (2003). The role of octadecanoids and functional mimics in soybean defense responses. Biol Chem.

[63] Taki N, Sasaki-Sekimoto Y, Obayashi T, Kikuta A, Kobayashi K, Ainai T, Yagi K, Sakurai N, Suzuki H, Masuda T, Takamiya K, Shibata D, Kobayashi Y, Ohta H (2005). 12-oxo-phytodienoic acid triggers expression of a distinct set of genes and plays a role in wound-induced gene expression in *Arabidopsis*. Plant Physiol.

[64] Mueller S, Hilbert B, Dueckershoff K, Roitsch T, Krischke M, Mueller MJ, Berger S (2008). General detoxification and stress responses are mediated by oxidized lipids through TGA transcription factors in Arabidopsis. Plant Cell.

[65] Ribot C, Zimmerli C, Farmer EE, Reymond P, Poirier Y (2008). Induction of the Arabidopsis PHO1;H10 gene by 12-oxo-phytodienoic acid but not jasmonic acid via a CORONATINE INSENSITIVE1-dependent pathway. Plant Physiol.

[66] Stotz HU, Mueller S, Zoeller M, Mueller MJ, Berger S (2013). TGA transcription factors and jasmonate-independent COI1 signalling regulate specific plant responses to reactive oxylipins. J Exp Bot.

[67] Li L, Zhao Y, McCaig BC, Wingerd BA, Wang J, Whalon ME, Pichersky E, Howe GA (2004). The tomato homolog of CORONATINE-INSENSITIVE1 is required for the maternal control of seed maturation, jasmonate-signaled defense responses, and glandular trichome development. Plant Cell.

[68] Eigenbrode SD, Trumble JT, Millar JG, White KK (1994). Topical toxicity of tomato sesquiterpenes to the beet armyworm and the role of these compounds in resistance derived from an accession of *Lycopersicon hirsutum f. typicum*. J Agr Food Chem.

[69] Roda AMY, Halitschke R, Steppuhn A, Baldwin IT (2004). Individual variability in herbivore-specific elicitors from the plant’s perspective. Mol Ecol.

[70] Dave A, Graham IA (2012). Oxylipin signalling: a distinct role for the jasmonic acid precursor *cis*-(+)-oxo-phytodienoic acid (*cis*-OPDA). Front Plant Sci.

[71] Park S-W, Li W, Viehhauser A, He B, Kim S, Nilsson AK, Andersson MX, Kittle JD, Ambavaram MMR, Luan S, Esker AR, Tholl D, Cimini D, Ellerström M, Coaker G, Mitchell TK, Pereira A, Dietz K-J, Lawrence CB (2013). Cyclophilin 20–3 relays a 12-oxo-phytodienoic acid signal during stress responsive regulation of cellular redox homeostasis. Proc Natl Acad Sci U S A.

[72] Goetz S, Hellwege A, Stenzel I, Kutter C, Hauptmann V, Forner S, McCaig B, Hause G, Miersch O, Wasternack C, Hause B (2012). Role of *cis*-12-Oxo-phytodienoic acid in tomato embryo development. Plant Physiol.

[73] Thaler JS, Farag MA, Paré PW, Dicke M (2002). Jasmonate-deficient plants have reduced direct and indirect defences against herbivores. Ecology Lett.

[74] Jongsma MA, Bakker PL, Peters J, Bosch D, Stiekema WJ (1995). Adaptation of *Spodoptera exigua* larvae to plant proteinase inhibitors by induction of gut proteinase activity insensitive to inhibition. Proc Natl Acad Sci U S A.

[75] Felton GW, Donato K, Del Vecchio RJ, Duffey SS (1989). Activation of plant foliar oxidases by insect feeding reduces nutritive quality of foliage for noctuid herbivores. J Chem Ecol.

[76] Stout MJ, Workman KV, Bostock RM, Duffey SS (1998). Stimulation and attenuation of induced resistance by elicitors and inhibitors of chemical induction in tomato (*Lycopersicon esculentum*) foliage. Ent Exp Appl.

[77] Bhonwong A, Stout MJ, Attajarusit J, Tantasawat P (2009). Defensive role of tomato polyphenol oxidases against cotton bollworm (*Helicoverpa armigera*) and beet armyworm (*Spodoptera exigua*). J Chem Ecol.

[78] Felton GW, Donato KK, Broadway RM, Duffey SS (1992). Impact of oxidized plant phenolics on the nutritional quality of dietary protein to a noctuid herbivore, *Spodoptera exigua*. J Insect Physiol.

[79] Constabel CP, Barbehenn RV, Schaller A (2008). Defensive roles of polyphenol oxidase in plants. Induced plant resistance to herbivory.

[80] Wang J, Constabel CP (2004). Polyphenol oxidase overexpression in transgenic *Populus* enhances resistance to herbivory by forest tent caterpillar (*Malacosoma disstria*). Planta.

[81] Cipollini DF, Redman AM (1999). Age-dependent effects of jasmonic acid treatment and wind exposure on foliar oxidase activity and insect resistance in tomato. J Chem Ecol.

[82] Cipollini D, Enright S, Traw MB, Bergelson J (2004). Salicylic acid inhibits jasmonic acid-induced resistance of *Arabidopsis thaliana* to *Spodoptera exigua*. Mol Ecol.

[83] Rayapuram C, Baldwin IT (2007). Increased SA in NPR1-silenced plants antagonizes JA and JA-dependent direct and indirect defenses in herbivore-attacked *Nicotiana attenuata* in nature. Plant J.

[84] Stotz HU, Koch T, Biedermann A, Weniger K, Boland W, Mitchell-Olds T (2002). Evidence for regulation of resistance in Arabidopsis to Egyptian cotton worm by salicylic and jasmonic acid signaling pathways. Planta.

[85] Weech MH, Chapleau M, Pan L, Ide C, Bede JC (2008). Caterpillar saliva interferes with induced *Arabidopsis thaliana* defence responses via the systemic acquired resistance pathway. J Exp Bot.

[86] Chung SH, Rosa C, Scully ED, Peiffer M, Tooker JF, Hoover K, Luthe DS, Felton GW (2013). Herbivore exploits orally secreted bacteria to suppress plant defenses. Proc Natl Acad Sci U S A.

[87] Bruessow F, Gouhier-Darimont C, Buchala A, Metraux JP, Reymond P (2010). Insect eggs suppress plant defence against chewing herbivores. Plant J.

[88] Sarmento RA, Lemos F, Bleeker PM, Schuurink RC, Pallini A, Oliveira MGA, Lima ER, Kant M, Sabelis MW, Janssen A (2011). A herbivore that manipulates plant defence. Ecol Lett.

[89] Lawrence SD, Novak NG, Blackburn MB (2007). Inhibition of proteinase inhibitor transcripts by *Leptinotarsa decemlineata* regurgitant in *Solanum lycopersicum*. J Chem Ecol.

[90] Chung SH, Felton GW (2011). Specificity of induced resistance in tomato against specialist Lepidopteran and Coleopteran species. J Chem Ecol.

[91] Lawrence S, Novak N, Ju C, Cooke J (2008). Potato, *Solanum tuberosum*, defense against Colorado potato beetle, *Leptinotarsa decemlineata* (Say): Microarray gene expression profiling of potato by Colorado potato beetle regurgitant treatment of wounded leaves. J Chem Ecol.

[92] Winz RA, Baldwin IT (2001). Molecular interactions between the specialist herbivore *Manduca sexta* (Lepidoptera, Sphingidae) and its natural host *Nicotiana attenuata*. IV. Insect-Induced ethylene reduces jasmonate-induced nicotine accumulation by regulating putrescine *N*-methyltransferase transcripts. Plant Physiol Biochem.

[93] Kahl J, Siemens DH, Aerts RJ, Gabler R, Kuhnemann F, Preston CA, Baldwin IT (2000). Herbivore-induced ethylene suppresses a direct defense but not a putative indirect defense against an adapted herbivore. Planta.

[94] Yang Z-W, Duan X-N, Jin S, Li X-W, Chen Z-M, Ren B-Z, Sun X-L (2013). Regurgitant derived from the tea Geometrid *Ectropis obliqua* suppresses wound-induced polyphenol oxidases activity in tea plants. J Chem Ecol.

[95] Opitz SW, Müller C (2009). Plant chemistry and insect sequestration. Chemoecology.

[96] Ode PJ (2006). Plant chemistry and natural enemy fitness: Effects on herbivore and natural enemy interactions. Annu Rev Entomol.

[97] Kumar P, Pandit SS, Steppuhn A, Baldwin IT (2014). Natural history-driven, plant-mediated RNAi-based study reveals CYP6B46’s role in a nicotine-mediated antipredator herbivore defense. Proc Natl Acad Sci U S A.

[98] Engelberth J, Seidl-Adams I, Schultz JC, Tumlinson JH (2007). Insect elicitors and exposure to green leafy volatiles differentially upregulate major octadecanoids and transcripts of 12-oxo phytodienoic acid reductases in *Zea mays*. Mol Plant Microbe Int.

[99] Zong N, Wang C-Z (2007). Larval feeding induced defensive responses in tobacco: comparison of two sibling species of *Helicoverpa* with different diet breadths. Planta.

[100] Tian D, Peiffer M, Shoemaker E, Tooker J, Haubruge E, Francis F, Luthe DS, Felton GW (2012). Salivary glucose oxidase from caterpillars mediates the induction of rapid and delayed-induced defenses in the tomato plant. PLoS ONE.

[101] Jones JDG, Dangl JL (2006). The plant immune system. Nature.

[102] Song W, Ma X, Tan H, Zhou J (2011). Abscisic acid enhances resistance to *Alternaria solani* in tomato seedlings. Plant Physiol Biochem.

[103] Bradford MM (1976). A rapid and sensitive method for the quantitation of microgram quantities of protein utilizing the principle of protein-dye binding. Anal Biochem.

